# Relative-to-human benchmark Cognitive Divergence and semantic comprehensibility in Chinese–Uyghur LLM translation

**DOI:** 10.3389/fpsyg.2026.1732609

**Published:** 2026-03-16

**Authors:** Jiaxin Zuo, Yiquan Wang, Xiadiya Yibulayin

**Affiliations:** 1 College of Chinese Language and Literature, Xinjiang University, Urumqi, China; 2 College of Mathematics and System Science, Xinjiang University, Urumqi, China

**Keywords:** Cognitive Divergence, cognitive load, dependency distance, Large Language Model, sentence processing

## Abstract

This study examines whether Large Language Models (LLMs) generate Chinese-to-Uyghur translations with syntactic patterns consistent with cognitive efficiency–motivated expectations. We compare translations produced by six mainstream LLMs with a benchmark generated by human experts and used for structural comparison. Syntactic complexity is quantified using Mean Dependency Distance (MDD), and we introduce a relative metric, Cognitive Divergence, as a structural proxy to capture sentence-level deviation from the human benchmark. Semantic comprehensibility is evaluated using COMET scores. The results indicate that LLM-generated texts show no statistically significant difference from the human benchmark in terms of macroscopic syntactic complexity, suggesting a form of surface-level syntactic similarity. However, absolute syntactic complexity alone does not exhibit a reliable association with semantic comprehensibility. In contrast, Cognitive Divergence shows a strong negative association with comprehensibility at the model level (*r* = −0.908, *p* = 0.012) and for most models at the sentence level. These findings suggest that relative alignment with human syntactic patterns may offer a useful explanatory perspective for understanding variation in the comprehensibility of LLM-generated translations, complementing existing evaluation approaches based on absolute complexity.

## 1 Introduction and related work

### 1.1 The challenge of evaluating LLM-generated text

For decades, the assessment of text complexity has often relied on formulaic, surface-level readability metrics such as the Flesch Reading Ease (FRE) index and the Gunning Fog (GF) index (Flesch, 1948; Gunning, 1952). These traditional formulas primarily operationalize difficulty through word- and sentence-length proxies, which may be informative at a coarse level but can overlook structural properties of syntax that are known to affect human processing. As Large Language Models (LLMs) increasingly generate fluent text, the limits of purely surface-based measures have become more visible, motivating renewed attention to cognitively grounded approaches to evaluation. For instance, Zang et al. (2024), in proposing the Language Predictability Score (LPS), argue that conventional readability formulas can be inadequate when they disregard linguistic context and the cognitive characteristics of the target audience. Together, these developments point to the value of evaluation paradigms inspired by psycholinguistics and cognitive science—particularly those that can help explain, through structural proxies, why texts with comparable surface characteristics may nonetheless differ in human comprehensibility.

Among cognitive principles relevant to language processing, Dependency Length Minimization (DLM) has been widely discussed as a mechanism that reduces cognitive load and supports processing fluency (Futrell et al., 2015). DLM posits a tendency for syntactically related constituents to remain relatively close in linear order during both production and comprehension, thereby limiting working-memory demands (Gibson, 1998). Consistent with this view, psycholinguistic evidence suggests that longer dependency distances are associated with increased processing costs, including longer reading times (Gildea and Temperley, 2010) and greater working-memory burden (Sagarra, 2017). This established link between syntactic structure and cognitive cost motivates examining whether and how LLM translation outputs differ from expert human translations in dependency-based properties, and how such differences relate to semantic comprehensibility in translation. While these principles inform our approach, we use dependency-based metrics as structural proxies to explore potential associations with processing fluency, rather than as direct measures of cognitive load.

### 1.2 Related work and motivation

Recent research on machine translation (MT) evaluation has advanced rapidly, particularly in response to modern neural MT systems and LLM-based translation. Broadly, prior work can be grouped into two complementary streams. The first stream focuses on metric meta-evaluation and system ranking through community-wide shared tasks, where benchmarks such as WMT provide authoritative evidence that MT evaluation remains an evolving, community-standard problem and that newer generation paradigms can challenge the assumptions underlying traditional metrics (Freitag et al., 2024; Lavie et al., 2025). Within this stream, neural evaluation metrics such as COMET have emerged as strong baselines due to their demonstrated correlation with human judgments (Rei et al., 2020). In parallel, related efforts have explored how LLMs can be used to improve translation outputs through systematic refinement procedures, such as TEaR. While effective for optimization, these approaches primarily aim to enhance output quality and do not explicitly provide interpretable, cognitively grounded indicators of why certain outputs may be easier or harder for humans to process (Feng et al., 2025).

The second stream investigates linguistic and cognitive properties of LLM outputs, often by characterizing absolute properties of generated texts, including readability measures or absolute syntactic complexity descriptors such as Mean Dependency Distance (MDD), to analyze human processing difficulty and translation behavior (Zang et al., 2024; Rashid et al., 2024; Jiang, 2025). While these approaches have yielded valuable insights, they tend to focus on absolute descriptors of text complexity rather than on sentence-aligned comparisons with expert human translations. As a result, fewer studies have operationalized the degree of syntactic alignment between LLM outputs and a human benchmark under the same source sentence as an explanatory factor for differences in semantic comprehensibility. Table 1 summarizes representative recent studies and situates the perspective adopted in the present work.

**Table 1:** Comparative analysis of recent literature on MT/text evaluation.

Study (year)	Core idea/metric	Strength	Limitation that motivates this paper
Freitag et al. (2024)	WMT24 metrics shared task; MT metric meta-evaluation	Authoritative shared-task evidence; establishes community standards under LLM-era conditions	Emphasizes metric validation and system ranking rather than cognitively interpretable mechanisms such as sentence-aligned syntactic alignment to a human benchmark
Lavie et al. (2025)	WMT25 shared task report; evaluation task framing and datasets	Defines community-wide evaluation settings, datasets, and baselines across languages	Focuses on system-level metric comparison, not on human-benchmark-relative syntactic alignment linked to comprehensibility
Zang et al. (2024)	Language Predictability Score (LPS) for readability	Cognitively grounded critique of traditional readability formulas; highlights contextual and audience effects	Targets corporate disclosure readability rather than translation; does not operationalize sentence-aligned alignment to an expert human reference
Li et al. (2025)	Evaluation of WMT25 metrics on SSA-MTE (African languages)	Highlights evaluation challenges in low-resource and linguistically diverse settings	Examines metric robustness but does not propose a cognitively grounded, human-benchmark-relative syntactic alignment measure
Rashid et al. (2024)	Readability comparison of LLM- and human-generated educational texts	Demonstrates that LLM-generated text can match or exceed human readability in specific contexts	Provides empirical comparison without an explanatory metric for when and why comprehensibility differences arise
Feng et al. (2025)	TEaR (Translate–Estimate–Refine) for LLM-based MT	Improves translation quality through iterative self-refinement	Optimization-oriented framework; lacks an interpretable, cognitively grounded indicator of sentence-level alignment to a human benchmark
Jiang (2025)	Dependency-grammar-based analysis of LLM translation (e.g., MDD)	Applies dependency-based absolute complexity measures to study LLM translation patterns	Relies on absolute descriptors and does not quantify sentence-aligned deviation from an expert human reference
Our study	Cognitive Divergence (relative-to-human benchmark)	Operationalizes sentence-aligned syntactic alignment to a human expert benchmark and links it to semantic comprehensibility	Directly tests whether deviation from human patterns, rather than absolute complexity alone, shows a stronger association with lower comprehensibility

Against this background, the present study introduces Cognitive Divergence as a structural proxy. This relative-to-human-benchmark measure quantifies how an LLM’s syntactic choices deviate from those of an expert human translation under the same source sentence. Rather than proposing an alternative evaluation metric for system ranking, our goal is to examine whether such relative syntactic deviation is systematically associated with semantic comprehensibility in LLM-based translation, and whether it offers complementary explanatory value beyond absolute complexity measures. Cognitive Divergence is conceptualized as a structural proxy inspired by cognitive efficiency principles, not as a direct empirical indicator of human cognitive processing.

### 1.3 The present study

Motivated by this perspective and building on the comparative framework summarized in Table 1, the present study formulates three research questions to examine syntactic complexity, human-benchmark-relative divergence, and their relationships with semantic comprehensibility in LLM-based translation:

(1) Is there a significant difference in the absolute syntactic complexity (MDD) of texts generated by different LLMs and the human expert benchmark?
(2) What is the relationship between the absolute syntactic complexity (MDD) of LLM-generated texts and their semantic comprehensibility?
(3) What is the association between the Cognitive Divergence of LLM-generated texts from the human benchmark and their semantic comprehensibility?

## 2 Materials and methods

### 2.1 Theoretical basis

The experimental design and metric construction of this study are inspired by two core theories from psycholinguistics.

#### 2.1.1 Dependency length minimization and sentence processing

Dependency Grammar (DG) represents syntactic structure by modeling head–dependent relations between words (Tesnière, 1959). Within this framework, the linear distance between two words directly connected by a syntactic dependency is referred to as Dependency Distance (DD) (Hudson, 1995; Temperley, 2007). Aggregated dependency-distance measures have been widely used as quantitative proxies for syntactic complexity in sentence processing research (Ferrer-i-Cancho, 2004; Liu et al., 2009; Liu, 2008; Oya, 2011).

More importantly, dependency distance has been widely discussed in relation to human sentence-processing demands. Large-scale corpus studies, such as Futrell et al. (2015), provide quantitative evidence for this tendency, suggesting that Dependency Length Minimization (DLM) is widely observed across languages. This tendency is often interpreted as reflecting general cognitive constraints on memory and integration during sentence processing, consistent with efficiency-based accounts of language use. Related work has argued that dependency-length pressures may contribute to diachronic change in language use (Lei and Wen, 2020).

The classic explanation for this phenomenon comes from Gibson’s (1998, 2000) Dependency Locality Theory (DLT). This theory posits that sentence-processing difficulty reflects both storage and integration costs, and that integration cost increases with the distance over which dependencies must be integrated. Evidence from cognitive neuroscience further supports associations between syntactic complexity and processing demands. For example, studies suggest that processing sentences with greater syntactic complexity is associated with increased engagement of language-related neural networks, including regions often linked to syntactic processing such as Broca’s area (Friederici and Brauer, 2009; Umejima et al., 2023). Such findings are consistent with the view that higher syntactic complexity can place greater demands on cognitive resources (Yang et al., 2017; Phillips et al., 2005; Zhang et al., 2024). These theories provide a cognitive motivation for our structural metrics, but our analyses rely on syntactic proxies rather than direct cognitive experimentation.

#### 2.1.2 Cognitive load and working memory

The conservation of cognitive resources by the DLM principle can be further explained by Cognitive Load Theory (CLT) (Sweller, 1988). In the context of language comprehension, the syntactic structure of a sentence is one of the key factors constituting intrinsic cognitive load (Paas and Van Merriënboer, 1994). A sentence with a complex syntactic structure and numerous long-distance dependencies requires readers to simultaneously store and process multiple unclosed syntactic dependency arcs in their limited working memory. A systematic review of fMRI studies over the past two decades indicates that the engagement of the brain’s working memory system is dynamically increased with the complexity of language tasks, and its activation areas partially overlap with those for processing syntactic complexity (Sagarra, 2017; Deldar et al., 2020). Therefore, within the framework of this study, human expert translations are treated as a reference shaped by institutional norms and genre-specific constraints, serving as a benchmark for structural comparison rather than a direct measure of universal cognitive optimality. This reference reflects translation choices that have been shaped by practical constraints on accuracy and processing fluency in a given institutional context.

### 2.2 Corpus and preprocessing

This study selected the full official Chinese text of the “Report to the 20th National Congress of the Communist Party of China” as the source corpus. We used the official Uyghur translation as the human expert benchmark. This benchmark text was sourced from the officially published book, Guidance Reader for the Report to the 20th CPC National Congress (Uyghur Edition) (ISBN: 978-7-105-16886-6), which was translated and published by the Ethnic Publishing House under the auspices of the China National Ethnic Language Translation Bureau. The selection of this official text is justified by its rigorous, multi-stage translation and review process, which aims to ensure clarity, accuracy, and stylistic appropriateness for the target audience, consistent with established norms observed in the translation of political discourse across languages (Valdeón and Li, 2024; Zhao and Wang, 2025). Such expert-driven revision practices are also described in institutional accounts of Uyghur political translation (Ablimit and Wuqiman, 2022). According to published accounts, this process involves certified senior translators who are native speakers and experts in the subject matter, followed by meticulous cross-checking and review by an editorial committee. For the purposes of this study, the human expert benchmark is operationalized as a specific institutional reference rather than a universal cognitive norm. We acknowledge that such translations are shaped by institutional constraints and genre-specific norms, which do not necessarily reflect general cognitive optimality or naturalistic language production. Nevertheless, the rigorous multi-stage expert revision workflow prioritizes clarity and readability for the target readership. This procedure allows the text to serve as a practical structural baseline within this specific domain.

The corpus preprocessing pipeline was executed using Python (version 3.13.2) with the pandas library (version 2.3.2). The initial step involved data cleaning, where all non-textual elements, such as headers, footers, and page numbers, were programmatically removed from the source documents. Subsequently, the cleaned text was segmented into individual sentences based on punctuation marks (e.g., periods, question marks, exclamation points). This process yielded a total of 644 sentences. To characterize the distribution of sentence lengths in the corpus and support subsequent sampling, we conducted a statistical analysis of the lengths of all sentences.

To ensure the sample was representative across different levels of sentence complexity, a stratified sampling scheme was designed based on quartile statistics. All 644 sentences were divided into three strata: short sentences (≤22 characters, 166 sentences), medium-to-long sentences (22–65 characters, 318 sentences), and long sentences (>65 characters, 160 sentences). Subsequently, a random sample of approximately 33% was drawn from each stratum, ultimately forming a source sample set of 213 sentences.

These 213 sentences were then fed into six Large Language Models for the translation task. The specific model versions used were: Gemini-2.5 Flash, DeepSeek-V3.1, ChatGPT-5, Kimi K2-0905, Qwen3-Max-Preview, and Hunyuan-Turbo-S (All model tests were completed in September 2025 to ensure version consistency). To control for variables, a unified prompt was used. The Chinese prompt “请将以下句子翻译成维吾尔语，每句单独成行” (Please translate the following sentences into Uyghur, with each sentence on a separate line) was used for the four domestic models, while the English prompt “Please translate the following sentences into Uyghur, with each sentence on a separate line” was used for the other two foreign models. The translations were obtained by inputting the prompts and source sentences directly into the web-based conversational interface of each model. After obtaining all generated texts, a final data cleaning step was performed. This involved a manual inspection to remove any empty lines, conversational artifacts (e.g., “Here is the translation:”), headers, or footers, without altering the linguistic content of the translations, ensuring that only the core translated text remained in the individual corpus for each LLM.

### 2.3 Metrics and tools

#### 2.3.1 Mean dependency distance

Mean Dependency Distance (MDD) is a classic metric used to measure the syntactic complexity of a sentence. It is built upon the framework of Dependency Grammar. In a sentence, the linear distance between any two words directly connected by a dependency relation (i.e., a head and its dependent) is called Dependency Distance (DD). This distance is typically calculated by the number of words separating the two words; for instance, in the Uyghur sentence “u alma yedi” (He ate an apple), if the head verb “yedi” (ate) and its subject “u” (he) are separated by one intervening word, the dependency distance between them is counted as 2.

The MDD of a sentence is the sum of all its dependency distances divided by the total number of dependency relations. The formula for its calculation is as follows:


$$MDD\left(thesentence\right)=\frac{1}{n-1}\overset{n-1}{\underset{i=1}{\sum }}\left|DD_{i}\right|$$

Here, *n* represents the total number of words in the sentence, making the total number of dependency relations *n* − 1. To ensure computational accuracy, all punctuation marks and the virtual ROOT node are excluded from the calculation.

Within the cognitive framework of this study, MDD is operationally defined as an indicator related to cognitive processing cost. According to DLT and CLT, a higher MDD in a sentence is associated with a looser overall syntactic structure and is generally linked to increased intrinsic cognitive load (Gibson, 2000; Sweller, 1988). Accordingly, higher MDD values are interpreted as indicating lower levels of cognitive friendliness for the reader.

To ensure objectivity and replicability, the entire process of identifying dependency relations and calculating MDD was fully automated, not based on manual evaluation. All dependency parsing and MDD calculations were performed by calling the Stanza toolkit (version 1.10.1) from Stanford University (Qi et al., 2020) through Python programming. The specific processor chain for Uyghur (ug) utilized the udt_nocharlm model package, which includes tokenization, part-of-speech tagging, lemmatization, and dependency parsing (tokenize, pos, lemma, depparse). Within this framework, the term “directly connected” must be understood in its specific technical sense: it refers exclusively to syntactically linked head-dependent pairs in the dependency parse tree, not to broader semantic associations.

#### 2.3.2 Cognitive Divergence

To measure the extent to which machine-generated syntactic patterns deviate from the human expert reference, this study introduces a relative evaluation metric termed “Cognitive Divergence.” It is crucial to explicitly clarify that this metric functions as a structural proxy inspired by cognitive theory, rather than a direct empirical measure of human cognition or cognitive load. It is defined as the absolute difference between the MDD of a single sentence generated by an LLM and the MDD of the corresponding single sentence in the human-expert-generated benchmark:


$$\mathrm{Cognitive Divergence}=\left|\begin{array}{l} MDD\left(LLM-generatedsentence\right)\\ -MDD\left(Human-expertsentence\right) \end{array}\right|$$

A key assumption, grounded in psycholinguistics, is that human expert translations can be viewed as reflecting a “good-enough” processing solution that balances expressive needs with cognitive efficiency (Cong and Rayz, 2025). Thus, Cognitive Divergence measures deviation from this specific institutional reference rather than from a universal cognitive norm, providing a nuanced tool for assessing syntactic alignment in domain-specific translations. Therefore, we hypothesize that a smaller Cognitive Divergence value indicates that the LLM’s syntactic choices more closely align with human cognitive habits. Conversely, a larger divergence, regardless of whether the resulting sentence is “simpler” or “more complex” in absolute terms, is expected to be associated with increased cognitive friction for readers, as it departs from familiar processing patterns. This metric thus shifts the focus from “absolute complexity” to “relative deviation,” providing a more nuanced tool for assessing the cognitive alignment between LLM output and human language patterns.

#### 2.3.3 COMET score

To objectively evaluate the comprehensibility of LLM-generated texts from a semantic perspective, this study employed the automated evaluation tool COMET, which is used as a widely adopted proxy indicator for semantic comprehensibility. Unlike traditional metrics such as BLEU or METEOR that rely on surface-level n-gram overlap, COMET is a neural-based metric that leverages large pre-trained language models to assess the semantic similarity between a machine translation, its source text, and a human reference (Rei et al., 2020). This deep-learning approach allows it to capture semantic nuances more effectively. Indeed, recent benchmarking studies have consistently shown that newer, neural-based metrics like COMET have a significantly higher correlation with human judgments of translation quality compared to their predecessors (López Caro, 2024). Therefore, following this best practice, this study specifically utilized the high-performing wmt22-comet-da model released by Unbabel (Freitag et al., 2022), where a higher score signifies higher text comprehensibility.

#### 2.3.4 Statistical analysis

All statistical analyses were conducted using Python (version 3.13.2) and its scientific computing ecosystem to ensure transparency and reproducibility.

To examine differences in syntactic complexity across translation sources, a one-way analysis of variance (ANOVA) was performed on sentence-level Mean Dependency Distance (MDD) values, with translation source (the human expert benchmark and six LLMs) as the grouping factor. In this analysis, each translated sentence constituted one observation, and sentences were grouped according to their translation source. The ANOVA was implemented using the stats.f_oneway function from the scipy.stats module (version 1.16.1). The resulting ANOVA summary statistics are reported in Table 2.

**Table 2:** Analysis of variance (ANOVA) for Mean Dependency Distance across human benchmark and LLM-generated texts.

Source of variation	Degrees of freedom	Sum of squares	Mean square	*F*	*p*
Between groups	6	11.747017	1.9679	2.1847	0.042*
Within groups	1,484	1336.7168	0.9008		

The “*” indicates statistical significance at the *p* < 0.05 level.

Although translations produced by different systems were derived from the same set of source sentences, treating sentence-level MDD values as observations grouped by translation source follows common practice in large-scale syntactic complexity analyses of translated text, where the primary interest lies in distributional differences across systems rather than sentence-by-sentence paired comparisons.

When the overall ANOVA indicated a significant main effect, post-hoc multiple comparisons were conducted using Tukey’s Honestly Significant Difference (HSD) test, as implemented in the statsmodels.stats.multicomp module (version 0.14.5), to identify pairwise differences between the human benchmark and individual LLMs.

All statistical procedures were implemented programmatically to ensure replicability.

## 3 Results

### 3.1 Syntactic complexity characteristics of the corpus

**Figure 1:**
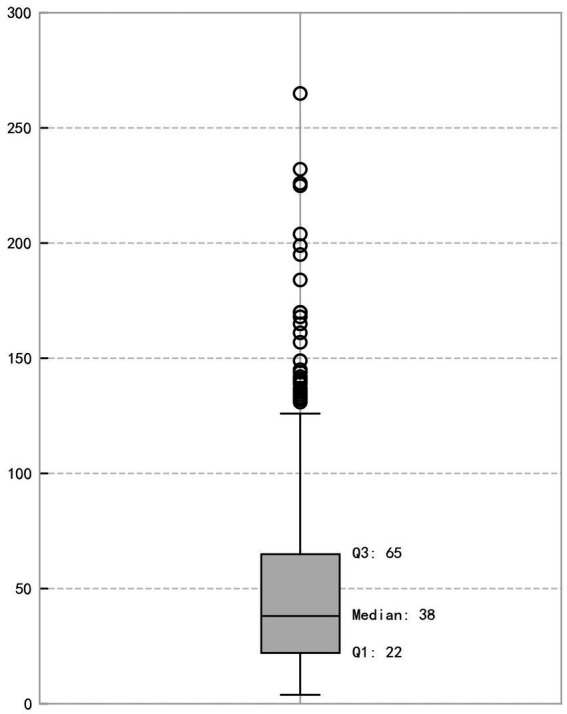
Boxplot of sentence lengths for the source corpus (*n* = 644).

The analysis of sentence lengths for the source corpus (*n* = 644) is shown in Figure 1. The boxplot clearly illustrates the distribution: the median length is 38 characters, with the first quartile (Q1) at 22 and the third quartile (Q3) at 65. The data exhibit a significant right-skewed distribution. This distributional pattern is commonly observed in natural language corpora, where short and medium-length sentences are more frequent than very long ones, reflecting general regularities in language use (Zipf, 1949). The long tail of this distribution, which contains a substantial number of structurally complex long sentences, underscores the necessity of our stratified sampling approach. Relying on simple random sampling would likely result in an underrepresentation of these challenging sentences, which are crucial for testing the limits of both human and machine processing capabilities. Our three-stratum design ensures that each level of complexity is adequately represented in the final sample, thus enhancing the validity of our subsequent analyses.

### 3.2 Syntactic complexity of LLM-generated texts

We performed dependency parsing on the source texts, the human expert benchmark, and the texts generated by the six Large Language Models (*n* = 213 sentences each) to calculate their Mean Dependency Distance (MDD). Table 3 presents the MDD calculation results for a selection of individual sentences, while Table 4 summarizes the overall mean MDD for each text type.

**Table 3:** Mean Dependency Distance (MDD) for individual Chinese-to-Uyghur translated sentences generated by Large Language Models.

Sentence	Text source	MDD
1	Source text (Chinese)	2.833333333
	Human expert benchmark	1.555555556
	Gemini	2.153846154
	ChatGPT-5	2.5
	DeepSeek	2
	Kimi	2.444444444
	Qwen	1.571428571
	Hunyuan	3.333333333
…		
213	Hunyuan	2.723404255

**Table 4:** Overall Mean Dependency Distance (MDD) of texts from different sources.

Text source	Mean Dependency Distance (MDD)
Source text (Chinese)	3.686227771
Human expert benchmark	2.954545455
Gemini	2.845842356
ChatGPT-5	2.623703226
DeepSeek	2.739736672
Kimi	2.769723836
Qwen	2.873939248
Hunyuan	2.688282656

To test for significant differences in MDD across the various text sources, a one-way analysis of variance (ANOVA) was conducted. The results (see Table 2) show a statistically significant difference in the mean MDD across the seven text versions (*F*(6, 1,484) = 2.18, *p* = 0.042). However, the results of the Tukey HSD post-hoc multiple comparison test indicated that there were no statistically significant differences between the mean MDD of any LLM-generated text and that of the human expert benchmark (all *p* > 0.05).

### 3.3 Correlation analysis: Cognitive Divergence as a stronger correlate of comprehensibility

A core objective of this study was to determine which metric—traditional syntactic complexity (MDD) or our proposed Cognitive Divergence—serves as a more reliable indicator of semantic comprehensibility in LLM-generated translations. To investigate this, we first used the wmt22-comet-da model to score all generated texts, obtaining a proxy indicator for their semantic comprehensibility. Table 5 provides a sample of these scoring results for several individual sentences, illustrating the variance in quality across different models. The overall mean scores for each LLM are presented alongside their MDD scores in Table 6.

**Table 5:** Semantic Comprehensibility (COMET) scores for individual Chinese-to-Uyghur translated sentences generated by Large Language Models.

Sentence	Text source	Comprehensibility score
1	Human expert benchmark	1
	Gemini	0.896354258
	ChatGPT-5	0.790922403
	DeepSeek	0.883804142
	Kimi	0.880448878
	Qwen	0.821790516
	Hunyuan	0.904007852
…		
213	Hunyuan	0.859381557

**Table 6:** Overall MDD and COMET scores for each LLM.

Text source	Mean Dependency Distance (MDD)	Overall comprehensibility score
Human expert benchmark	2.645495	1
Gemini	2.845842356	0.890737483
ChatGPT-5	2.623703226	0.818213213
DeepSeek	2.739736672	0.838196611
Kimi	2.769723836	0.799825964
Qwen	2.873939248	0.725821855
Hunyuan	2.688282656	0.755580976

#### 3.3.1 The relationship between traditional metrics (MDD) and comprehensibility

First, we examined the relationship between syntactic complexity (MDD) and semantic comprehensibility (COMET scores) using the macro-level data from Table 6. A Pearson correlation analysis showed a non-significant correlation (*r* = −0.023, *p* = 0.965). To further validate this, a Spearman rank correlation test was also performed, which yielded a similarly non-significant result (*rₛ* = −0.143, *p* = 0.787). Both tests consistently indicate that, within our dataset, a significant statistical relationship between lower syntactic complexity (MDD) and higher semantic comprehensibility could not be established. These results indicate that, within the present dataset, no statistically significant association was observed between lower MDD values and higher semantic comprehensibility scores.

#### 3.3.2 The relationship between the proposed metric (Cognitive Divergence) and comprehensibility

**Figure 2:**
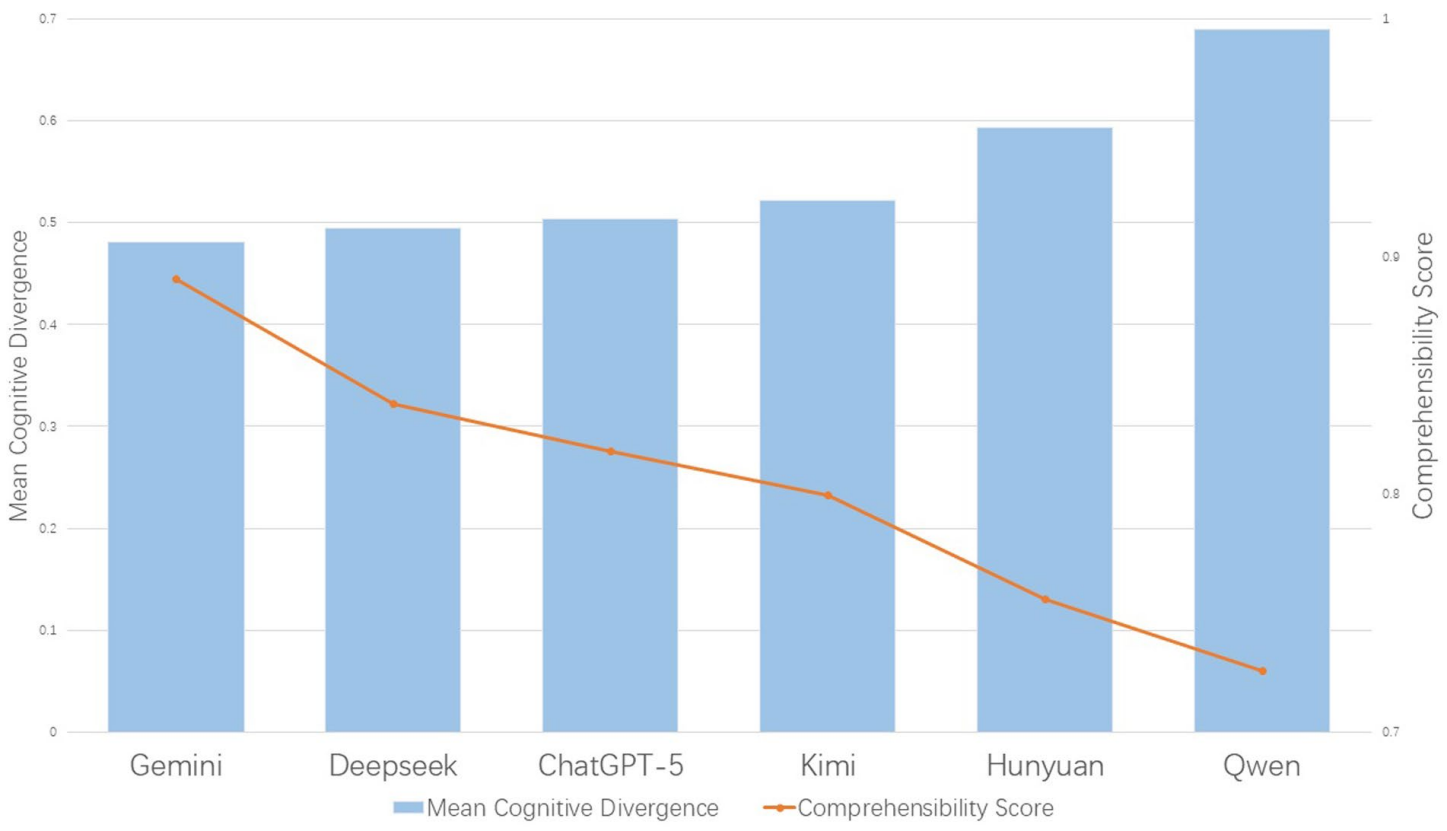
Correlation between mean Cognitive Divergence and COMET scores.

Next, we examined the relationship between the proposed metric, Cognitive Divergence, and semantic comprehensibility. At the macro level, using the model-level mean values reported in Table 6, a Pearson correlation analysis revealed a strong negative association between Cognitive Divergence and COMET scores within the evaluated systems (*r* = −0.908, *p* = 0.012). This relationship is illustrated in Figure 2, indicating that models whose syntactic structures more closely aligned with the human expert benchmark tended to achieve higher semantic comprehensibility scores.

Given the limited number of models included in the analysis (*N* = 6), a supplementary Spearman rank correlation test was conducted to assess the robustness of this finding. The Spearman analysis likewise revealed a strong negative monotonic relationship (*rₛ* = −1.00, *p* < 0.001), suggesting that the observed association does not depend on strict linear assumptions within the set of evaluated models.

To further explore whether this pattern extends beyond aggregate model-level trends, we conducted sentence-level correlation analyses for each LLM separately. As shown in Table 7, five of the six models exhibited statistically significant negative correlations between Cognitive Divergence and COMET scores, indicating that greater deviation from the human benchmark at the sentence level was generally associated with lower semantic comprehensibility. One exception was ChatGPT-5, for which the sentence-level correlation did not reach statistical significance. This suggests that, for this model, higher syntactic divergence from the human benchmark does not necessarily coincide with reduced semantic comprehensibility.

**Table 7:** Correlation analysis between “Cognitive Divergence” and semantic comprehensibility (COMET) scores.

Text source	Pearson correlation (*r*)	*p*-value	Significance
Gemini	−0.1687	0.0137	Yes
ChatGPT-5	−0.0150	0.8280	No
DeepSeek	−0.2005	0.0033	Yes
Kimi	−0.2313	0.0007	Yes
Qwen	−0.2088	0.0022	Yes
Hunyuan	−0.4188	<0.0001	Yes

Overall, these results indicate that Cognitive Divergence provides a more consistent association with semantic comprehensibility than absolute syntactic complexity (MDD) within the present dataset. Rather than implying that lower complexity is inherently preferable, the findings suggest that relative alignment with human expert syntactic patterns may offer a useful explanatory perspective when assessing LLM-generated translations.

## 4 Discussion

### 4.1 Surface-level syntactic similarity and its explanatory limits

One important observation of this study is that current mainstream Large Language Models (LLMs), when performing Chinese-to-Uyghur translation, can produce outputs whose macroscopic syntactic complexity, as measured by Mean Dependency Distance (MDD), is comparable to that of human expert translations, with no statistically significant differences observed between them (see Table 2). This result suggests that LLMs are capable of approximating overall syntactic complexity patterns of the target language within a specific translation context, a phenomenon we refer to as surface-level syntactic similarity.

However, the present findings also indicate that such surface-level similarity does not necessarily correspond to comparable levels of semantic comprehensibility. Specifically, no statistically significant relationship was observed between absolute syntactic complexity (MDD) and semantic comprehensibility (*r* = −0.023, *p* > 0.05; see Section 4.3). This suggests that sentences with lower syntactic complexity are not automatically easier to comprehend, and that reduced dependency distance alone may not adequately capture the factors that contribute to human understanding.

Taken together, these results imply that matching macroscopic syntactic features on a statistical level may be insufficient, by itself, to account for variation in comprehensibility among LLM-generated texts. Consequently, additional perspectives beyond absolute syntactic complexity are needed to better explain why texts with similar surface characteristics can differ substantially in how easily they are processed by human readers.

### 4.2 “Cognitive Divergence” as a key structural correlate of comprehensibility

In this context, it is important to clarify why Cognitive Divergence is defined using the absolute difference from the human benchmark. Although lower Mean Dependency Distance is often intuitively associated with reduced processing difficulty, in a translation setting, an MDD value that is substantially lower than the expert reference may also signal over-simplification or the loss of essential syntactic relations. For example, excessive omission of functional elements such as prepositions or conjunctions can reduce dependency distance while simultaneously disrupting semantic coherence. Therefore, the use of absolute deviation is intended to serve as a structural proxy for two symmetric sources of potential cognitive difficulty: excessive syntactic complexity that overloads working memory, and excessive structural simplification that undermines semantic completeness. From this perspective, both directions of deviation from the human benchmark represent departures from this specific institutional reference.

Through a specific case analysis of the Kimi model, we further examine how higher Cognitive Divergence may manifest at the sentence level. As shown in Table 8, the selected Kimi-generated sentence exhibits a substantially higher MDD value (MDD = 4.50) than the corresponding human expert translation (MDD = 2.33), indicating a looser syntactic structure.

**Table 8:** Case analysis of high Cognitive Divergence associated with low comprehensibility.

Category (MDD)	Text sample
Chinese source (MDD = 3.00)	只有植根本国、本民族历史文化沃土，马克思主义真理之树才能根深叶茂。(Only when the tree of Marxist truth is rooted in the fertile soil of the history and culture of its own country and nation can it grow deep roots and luxuriant leaves.)
Human benchmark (MDD = 2.33)	pɛqɛt dølitimiz vɛ millɛtimizniŋ tɑrixij mɛdɛnijɛt tupriqida jiltiz tɑrtquzʁɑndilɑ, mɑrksizm hɛqiqitidin ibɑrɛt bu. dɛrɛxni bɑrɑqsɑn ɑjnitɑlɑjimiz.
Kimi-generated text (MDD = 4.50)	pɛqɛt øz dølitiniŋ, øz millitiniŋ tɑrixij mɛdɛnijɛtlik toprɑqqɑ tʃiqip, mɑrksizm hɛqiqiti dɛrɛxiniŋ jiltizi tʃoŋqur, jɑprɑqi mol bolɑlɑjdu.

**Figure 3:**
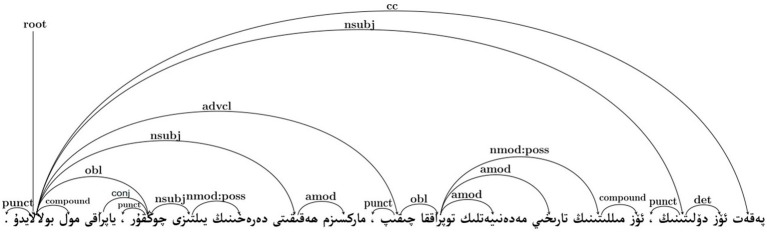
Dependency tree of the text generated by the Kimi model.

**Figure 4:**
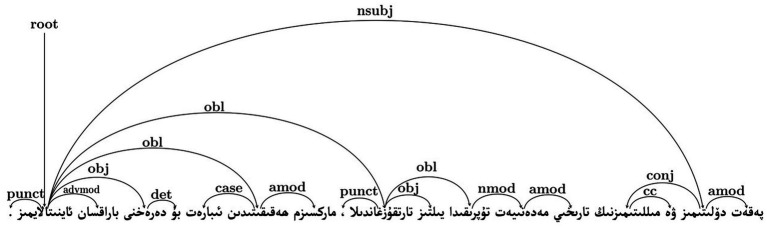
Dependency tree of the human benchmark text.

A comparison of the dependency trees in Figure 3 (human expert benchmark) and Figure 4 (Kimi output) reveals clear structural differences. In particular, the Kimi translation displays a displacement of the core predicate and a rigid transfer of source-language nominal structures, resulting in longer dependency arcs between syntactically related elements. Such configurations increase the distance over which dependencies must be integrated during sentence processing.

From a cognitive perspective, these structural characteristics are associated with higher processing demands, as longer dependency distances require readers to maintain more unresolved syntactic relations in working memory. This observation is consistent with predictions derived from Dependency Locality Theory and Cognitive Load Theory, which link increased dependency distance to greater integration cost. Similar effects of inefficient syntactic compression strategies on processing difficulty have also been noted in recent work by Isono (2024). Importantly, this example serves as an illustrative case, highlighting one structural pathway through which elevated Cognitive Divergence may be associated with reduced comprehensibility, rather than as definitive causal evidence.

### 4.3 “Similar in form, different in spirit”: the importance of syntacto-semantic integrity

An instructive observation emerges from the outlier analysis of ChatGPT-5 (see Table 9). Although the translation generated by this model exhibits very low Cognitive Divergence, indicating close syntactic alignment with the human benchmark (“similar in form”), its semantic comprehensibility score remains relatively low (“different in spirit”).

**Table 9:** Case analysis of comprehension failure despite low Cognitive Divergence.

Category (MDD)	Text sample
Chinese source (MDD = 3.13)	发挥领导干部示范带头作用，努力使尊法学法守法用法在全社会蔚然成风。(Give full play to the exemplary and leading role of leading cadres, and strive to make respecting, learning, abiding by, and using the law a common practice throughout society.)
Human benchmark (MDD = 4.17)	rɛhbiri kɑdirlɑrniŋ ylgilik bɑʃlɑmtʃiliq rolini dʒɑri qildurup, qɑnunʁɑ hørmɛt qiliʃ, qɑnunni øginiʃ, qɑnunʁɑ riɑjɛ qiliʃ, qɑnunni tɛtbiqlɑʃni tiriʃip pytkyl dʒɛmijɛtniŋ omumij kɛjpijɑtiʁɑ ɑjlɑnduruʃimiz kerɛk.
ChatGPT-5-generated text (MDD = 4.14)	rɛhbɛr kɑdirlɑrniŋ ørnɛk rolini toluq ojnitip, «qɑnunʁɑ hørmɛt-øginiʃ-sɑqlɑʃ-qollɑnmɑq» ni pytkyl dʒɑmɑɛttɛ ɛŋ jɑxʃi ɑdɛt qiliʃ.

Closer examination of the example in Table 9 suggests that this discrepancy may be associated with a disruption of syntacto-semantic integrity. Specifically, the omission of a core predicate results in a grammatically incomplete sentence, which can hinder readers from achieving full semantic interpretation and sentence-level closure. Rather than reflecting increased syntactic complexity, the reduced comprehensibility in this case appears to stem from structural incompleteness at a critical local level.

This observation indicates that minimizing Cognitive Divergence alone may be a necessary but not sufficient condition for producing highly comprehensible text. Once a model is able to approximate human-like syntactic patterns at a global level, limitations in local structural integrity can still undermine interpretation. Consequently, beyond achieving relative syntactic alignment, ensuring the grammatical completeness and semantic coherence of core sentence structures remains essential for human-oriented language generation.

### 4.4 Dialogue with related research and the uniqueness of this study

The results of this study contribute empirical observations to ongoing discussions in cognitive science regarding the extent to which statistical learning accounts for language complexity (Contreras Kallens et al., 2023).

Taken together, the present findings are consistent with prior work suggesting that, despite the effectiveness of LLMs in statistical pattern learning, certain efficiency-oriented properties of human language production may not be fully captured (Lakretz et al., 2020; Mahowald et al., 2024).

Interestingly, the patterns observed in the present study differ from those described by the “compromise hypothesis in translation studies (Fan and Jiang, 2023). The syntactic “compromise” feature exhibited by human translators—where the MDD of the translation lies between that of the source and target languages—may reflect a dynamic cognitive balance between “fidelity to the source structure” and “conformity to target language norms.” In contrast, the high degree of “superficial mimicry” shown by LLMs more closely resembles surface-level pattern reproduction, rather than reflecting the dynamic balancing processes described for human translators. This “similar in form, different in spirit” pattern highlights a fundamental limitation of current LLM-based translation, namely that statistical adequacy does not necessarily entail cognitive alignment.

Furthermore, the “Cognitive Divergence” we observed at the syntactic level appears to have corresponding manifestations in other linguistic dimensions. For instance, recent research has found that at the level of discourse strategy, humans tend to use shorter, more informal, and analogy-rich language, whereas LLMs tend to generate longer, more formal, and policy-like texts (Zeleke et al., 2025). This observation is broadly consistent with our findings at the syntactic level, suggesting that divergence from human cognitive efficiency principles may manifest not only at the level of global syntactic descriptors, but also across multiple linguistic layers, from sentence structure to discourse strategy.

### 4.5 Limitations and future directions

This study acknowledges several limitations that point toward important avenues for future research. First, we used only MDD as a single indicator of syntactic complexity. While MDD serves as a foundational proxy, cognitive load during sentence processing is a multifaceted phenomenon. To capture this richness, future research should develop a multi-dimensional syntactic complexity framework. This framework could incorporate a comprehensive suite of metrics, such as clausal complexity (e.g., nesting depth), information-theoretic metrics like surprisal, and specifically for head-final languages like Uyghur, the directionality of dependency arcs and types of syntactic relations. Integrating these dimensions would allow for a more granular characterization of the cognitive performance load, including both global integration cost and local processing peaks.

An additional limitation concerns the accuracy of dependency parsing for Uyghur, a relatively low-resource language. Although the Stanza toolkit provides an officially supported Uyghur pipeline, parsing performance for low-resource languages is generally lower than that for high-resource languages, which may introduce noise into MDD and Cognitive Divergence calculations. However, because all LLM outputs and the human benchmark were processed using the same parser under identical conditions, such noise is expected to affect all systems in a comparable manner. Future work should validate the robustness of the proposed metric using improved parsers or manually annotated dependency trees.

Second, this study relied on COMET as a proxy indicator for comprehensibility, which inevitably simplifies the complex relationship between syntax and semantics. As the Associate Editor noted, semantic interpretation is a deeply complex and potentially non-formalisable domain. For example, the concept of Quantum Semantics (Raikov, 2021) models meaning as inherently context-dependent and uncertain. Future studies may therefore integrate Cognitive Divergence with more advanced semantic modeling approaches or establish causal links through controlled psycholinguistic experiments, such as eye-tracking or EEG/ERP studies (Raikov, 2022). In addition, the macro-level correlation analyses were conducted at the level of translation systems, where the number of observations is necessarily limited by the number of evaluated models. Accordingly, these results are intended to illustrate general trends across systems rather than to support fine-grained statistical generalization. Importantly, these macro-level findings are complemented by sentence-level analyses involving a substantially larger number of observations, which provide converging evidence for the reported associations. Crucially, we must emphasize that demonstrating a correlation between a structural deviation metric and an automated evaluation score establishes neither a causal mechanism nor definitive proof that the metric captures human cognitive processing. The varying strengths of the observed statistically significant correlations indicate an association rather than direct explanatory power.

Third, this study raises a broader open question regarding user experience. Although Cognitive Divergence shows a consistent association with text comprehensibility, the relationship between cognitive efficiency and human subjective preference may not be linear. Recent evidence suggests that while humans can reliably distinguish AI-generated text, they do not always prefer human-authored content (Wang et al., 2025). Future research should therefore explore how cognitive efficiency and subjective preference interact, and how both dimensions can be jointly optimized in human-aligned language generation systems.

Finally, beyond evaluation, this research envisions a transition from a diagnostic tool to a prescriptive framework for LLM development. Moving from the current state of surface-level mimicry to a future state of deeper alignment remains an exploratory goal. One related possibility is that Cognitive Divergence could be operationalized as an auxiliary reward signal in Reinforcement Learning from Human Feedback (RLHF). However, given that the current empirical evaluation is limited to a single domain and language pair, we explicitly frame this application as an exploratory possibility requiring further validation across diverse linguistic contexts. By incorporating penalties for syntactic patterns that deviate from human cognitive constraints, future work may guide models toward “Cognitive Convergence,” ensuring that generated text is not only statistically probable but also cognitively friendly.

## 5 Conclusion

This study investigated the relationship between the syntactic structures of LLM-generated translations and their semantic comprehensibility. Our findings indicate that traditional syntactic complexity metrics, such as Mean Dependency Distance (MDD), show limited explanatory power for translation quality in the context of modern LLMs. In contrast, we introduced and empirically examined Cognitive Divergence as a structural proxy, which measures the deviation of an LLM’s syntactic choices from a human expert benchmark. Our results suggest that Cognitive Divergence is consistently negatively associated with semantic comprehensibility, indicating that closer alignment with human syntactic patterns tends to correspond to higher perceived translation quality.

In response to the editorial feedback, we wish to clarify the term “humanized” as used in this context. We define a “human-like” (or “human-aligned”) text not as a vague esthetic quality, but as a measurable property: the degree of alignment with the syntactic choices a human expert makes under the same translation conditions. This operational definition moves beyond subjective preference and provides a quantifiable target for AI development. As research by Harish et al. (2024) suggests, fine-tuning LLMs with reinforcement learning is a promising pathway to produce text that more closely resembles human language. Our Cognitive Divergence metric could potentially serve as an exploratory reward signal for such fine-tuning processes, pending further validation across diverse domains and language pairs beyond the single context evaluated here.

Furthermore, we can conceptually frame this as a measure of Cognitive Convergence. For interpretability, Cognitive Convergence can be viewed as the mathematical inverse of Cognitive Divergence; therefore, minimizing divergence is functionally equivalent to maximizing convergence. This dual perspective offers an alternative way to interpret and communicate the proposed metric within a broader theoretical context.

Crucially, this study advocates for a paradigm shift from the “Current State” of evaluation to a “Future State” of active optimization. While current LLMs achieve “surface-level mimicry” via statistical probability, the next generation of models must move toward “Cognitive Alignment.” We propose that Cognitive Divergence (or Convergence) might eventually transition from a post-hoc diagnostic tool to an intrinsic objective function. Specifically, as an exploratory direction for future work, developers could explore incorporating this metric as a reward function within reinforcement learning-based alignment pipelines (e.g., RLHF; Ouyang et al., 2022), including more recent preference-based optimization approaches (Rafailov et al., 2023). By formulating penalties for syntactic structures that are hypothesized, based on psycholinguistic evidence, to impose excessive cognitive load, we can guide models to spontaneously generate text that respects human working memory constraints.

In conclusion, this research contributes not only a novel metric for evaluating LLM translation quality but also a concrete roadmap for the future. It provides empirical evidence that aligning with human cognitive principles represents a promising direction for developing more effective, robust, and truly human-centric language generation systems.

## Data Availability

The original contributions presented in the study are included in the article/supplementary material, further inquiries can be directed to the corresponding author.
